# An Overview on Microfluidic Systems for Nucleic Acids Extraction from Human Raw Samples

**DOI:** 10.3390/s21093058

**Published:** 2021-04-27

**Authors:** Daniele Obino, Massimo Vassalli, Alberto Franceschi, Andrea Alessandrini, Paolo Facci, Federica Viti

**Affiliations:** 1 Institute of Biophysics, National Research Council, 16149 Genova, Italy; daniele.obino@ibf.cnr.it (D.O.); federica.viti@ibf.cnr.it (F.V.); 2 Centre for the Cellular Microenvironment, James Watt School of Engineering, University of Glasgow, James Watt South Building, Glasgow G128LT, UK; massimo.vassalli@glasgow.ac.uk; 3 Diagnostica Veterinaria La Lanterna S.n.c., 16149 Genova, Italy; info@lanterna.biz; 4 Nanoscience Institute, National Research Council, 41125 Modena, Italy; andrea.alessandrini@unimore.it; 5 Department of Physics, Informatics and Mathematics, University of Modena and Reggio Emilia, 41125 Modena, Italy

**Keywords:** lab-on-chip, LOC, nucleic acid extraction, microfluidics, solid-phase extraction, SPE

## Abstract

Nucleic acid (NA) extraction is a basic step for genetic analysis, from scientific research to diagnostic and forensic applications. It aims at preparing samples for its application with biomolecular technologies such as isothermal and non-isothermal amplification, hybridization, electrophoresis, Sanger sequencing and next-generation sequencing. Multiple steps are involved in NA collection from raw samples, including cell separation from the rest of the specimen, cell lysis, NA isolation and release. Typically, this process needs molecular biology facilities, specialized instrumentation and labor-intensive operations. Microfluidic devices have been developed to analyze NA samples with high efficacy and sensitivity. In this context, the integration within the chip of the sample preparation phase is crucial to leverage the promise of portable, fast, user-friendly and economic point-of-care solutions. This review presents an overview of existing lab-on-a-chip (LOC) solutions designed to provide automated NA extraction from human raw biological fluids, such as whole blood, excreta (urine and feces), saliva. It mainly focuses on LOC implementation aspects, aiming to describe a detailed panorama of strategies implemented for different human raw sample preparations.

## 1. Introduction

Nucleic acid (NA) diagnostics, often called molecular diagnostics, consist of measuring DNA or RNA to evaluate the presence of pathological conditions, such as genetic dysregulations or invading pathogens. Most of the developed techniques in this field include a nucleic acid amplification step (commonly enabled through polymerase chain reaction—PCR—or loop amplification mediated polymorphism—LAMP—methods), which relies on nucleic acid extraction from samples [1]. Traditional NA diagnostics require access to suitable laboratories, equipped with bulky and expensive instrumentation that typically requires skilled personnel to be operated.

One of the objectives in current biotechnological research consists of developing molecular diagnostic tools easy to use, needing low amounts of samples, and possibly not requiring fully equipped labs to be operated. The concept of ‘lab-on-a-chip’ (LOC) goes towards this direction. The term LOC refers to a class of devices combining one or multiple biomolecular steps, typically handled in equipped laboratories, within a single miniaturized system, based on integrated microfluidic circuits. LOC enabling technologies include microfluidics [2], microelectronics [3], photolithography and other fabrication techniques [4], chemical or physical methods for cell lysis [5] and for NA purification [6,7].

The small dimension of such tools brings numerous advantages: (1) cost efficiency, directly derived from small size, which intrinsically reduces reagents and samples volumes; (2) automation, due to a self-standing experimental pipeline not needing human intervention; (3) diagnostic speed, directly related to automated performance of lab activities and reduced volumes; (4) ease of use, also deriving from LOC automation, which should simplify laboratory processes; (5) portability, enabled by the small size of the device and the absence of most of constraints related to an equipped laboratory; (6) reaction robustness, because an enclosed ‘sample-in answer-out’ system reduces the risk of cross-contamination, preserves NA purity and stability and enhances results reproducibility; (7) workers’ safety enhancement, avoiding direct contact with potentially toxic molecules [8,9,10,11].

LOCs have been designed to accomplish many biomolecular analyses, including nucleic acid extraction from raw samples, such as whole blood, urine, saliva, stool, thus fostering the shift of NA diagnostics from traditional laboratory tests to rapid tests, externalized to the point-of-care (POC), that allow distribution of NA diagnostics over a larger territory, to facilitate the access in remote areas and from impaired patients [12].

A functional and efficient POC platform, as well as a portable device for any biomolecular investigation, must be equipped with an autonomous sample preparation method, which critically impacts the efficiency and usability of the downstream assay. Despite the NA extraction phase from raw samples is important to enable a full POC procedure, this step has been rarely considered in the translation from lab practices to LOC approaches [13,14].

LOCs, POC tests and microfluidic systems are recurrent topics in technology advancement, and these concepts have been widely treated in the literature. Recent examples include the following: Ayoib et al. [15] and Reinholt et al. [16], who focus on the most common NA isolation techniques; Park et al. [17], who provide a detailed explanation of chip structures and sample motion strategies; Jayamohan et al. [2], who describe the most recurrent post-processing techniques; Kong et al. [18], who provide details regarding production and use of lab-on-a-disk (LOD) systems; Xu et al. [19], who describe microfluidic approaches to isolate cell-free DNA; Cui et al. [20], who summarize the major microfluidic techniques for preparation of clinically relevant samples (whole blood, urine, saliva); Bruijns et al. [21], who describe forensic applications of LOCs. Nevertheless, an exhaustive overview of NA extraction approaches from human raw samples enabled on LOCs can be useful for scientists approaching the POC theme. In this context, the present review proposes a critical overview of existing microfluidic devices that automate NA extraction from raw human samples, which includes the choices of the NA capture method (isolation) and of a suitable chip design, along with pre-treatment (cell lysis) and post-processing (purification) strategies. The NA extraction stage is a key component of integrated portable systems, and it massively impacts the effectiveness of successive steps of molecular diagnostics.

## 2. Microfluidic Systems for NA Extraction

**Figure 1:**
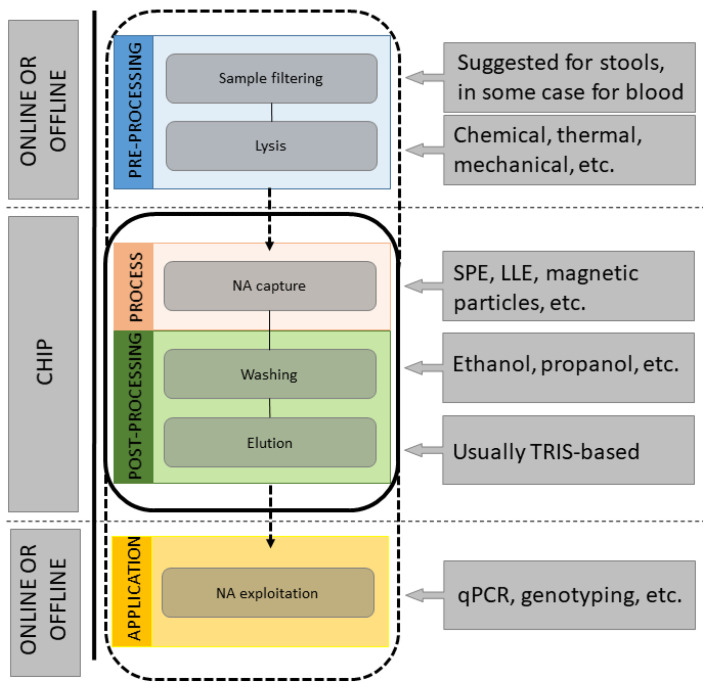
Theoretical scheme of a LOC for NA extraction from raw human samples. Raw samples are filtered, if necessary, and cells are lysed. NA are captured while other cellular components are removed. The successive step involves NA elution to obtain pure NA, which can be analyzed online or withdrawn for offline analyses.

Although many different LOCs have been developed in the last decades to perform this step, recurrent building schemes exist (Figure 1). The sample preparation pipeline includes different steps such as cell sorting, isolation and lysis, nucleic acids separation and collection. 

The diverse physical and chemical composition of the possible biospecimens (i.e., whole blood, urine, saliva, stool, etc.) does not allow for a universal separation module to be designed. Therefore, this review is organized on the basis of the different kinds of samples being fed to the device, rather than on the chemical/physical approach the extraction technology relies on, and on the extracted NA type.

Some of the described LOCs are developed with the only purpose of extracting NA for further analyses, while others are coupled with a subsequent amplification or detection steps in a continuous system. In this review, both of these LOC categories are considered, because the aim is to highlight methods for NA extraction useful for improving automation of existing amplification devices. Fostering the idea of POC, only LOCs for NA extraction from crude biological samplings are considered. A high number of automated, portable tools have been developed for extracting NA from whole blood. In fact, this biological sample, being usually taken from venous blood draw—but also easily with a fingerstick—is easily available especially for clinical analyses involving genomic or circulating DNA, or for investigating bacterial contaminations. Nevertheless, most of the devices initially developed for whole blood showed the capability of extracting NA even from other biological raw samples such as saliva, urine, semen, nasal swab, or spinal fluid.

Different solutions for NA extraction have been implemented in order to propose novel approaches and to obtain improved LOC performances in terms of shortening extraction time (while guaranteeing a reliable process) and reducing the need for large sample volumes (preserving the achievement of adequate NA concentration). A summary of these aspects is presented in Table 1, which lists characteristics of the LOCs presented in this review.

Considering the whole process from raw sample loading to NA collection, the described extraction times span from 7 to 50 minutes. Highly straightforward methods were proposed for NA extraction from blood and blood serum, presenting a purification time of about 1 minute. Although, depending on the extraction matrix properties and techniques, the minimum starting material needed for enabling NA successful extraction in LOCs is very small for whole blood (90 nL in Hung et al. [22]), serum (0.4 µL in Lee et al. [23]) and semen (1 µL in Bienvenue et al. [24]), while higher volumes appear to be needed for NA extraction from saliva (at least 10 µL in Legendre et al. [25]), urine (at least 50 µL in Han et al. [26]), or stool (at least 200 mg in Kang et al. [27]). Other considerations on reported features are discussed further in the text.

### 2.1. Microfluidic Circuits and Chip Structures

A crucial aspect in LOC development is chip structure. In general, an ideal solid surface made of a variable number of layers of elastomers, polymers, silicon or composite-glass is modeled with a pathway of microchannels and microchambers using different fabrication techniques, like micromachining, chemical etching, molding, embossing, laser ablation, soft lithography, and photolithography [77].

**Figure 2:**
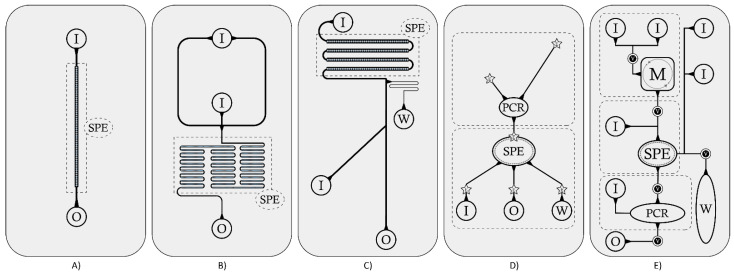
Schematization of chip structures from a simpler to a more complex design. (**A**) Chip with a simple linear structure: the main flux circulation is driven into a single microchannel through an inlet and an outlet port. The extraction domain consists of a part of microchannel with a packed adsorbent matrix polymerized inside it [10,23,24,46,75]. (**B**) Chip with a coil-shaped microchannel: the extraction domain is composed of a long microchannel with an increased surface/volume ratio of the adsorbent matrix packed inside it [31,42,61,69]. (**C**) Chip with a main microchannel and a side arm: sample and reagents are loaded in separate steps through different ports. Flux direction can be modulated at each phase by pressure application [9,25,33,37,76]. (**D**) Chip with electrokinetic motion: sample and pre-stored reagents are driven by voltage modulation into the extraction and amplification domains [65]. (**E**) Chip with a multi-domain design: each step of the sample processing is managed by valve activation. Generally, complex chips allow a whole sample treatment, from the lysis, the NA extraction with the development of various extraction techniques and, in some cases, the sample post-processing [27,35,39,44,47,66,67,71]. I = Sample or reagents inlet; O = Sample outlet; SPE = Solid-phase extraction domain; W = Waste chamber; PCR = Amplification chamber; E = Electrodes; V = Valves; M = Mixing or lysis chamber.

These systems of channels and chambers can be organized in domains or modules, each for a precise processing step of the sample in its fluidic circulation (Figure 2).

Microfluidics—defined as the use of micrometer scale channels to manipulate and process low volumes (10^−9^ to 10^−18^ L) of fluid samples [78]—is a crucial concept in LOC development. Microfluidic strategy consists in the controlled passage of fluids through the microchannels patterned on the chip, enabling the sample to be transferred from the inlet port to the elution chamber, passing through capture and collection steps. Moving through LOCs, fluids are suitably directed, mixed, separated or manipulated to attain the desired automated steps. The network of microchannels incorporated into the chip is connected to the external environment by drilled holes of different size; through these pathways, samples are injected into and evacuated from the microfluidic chip.

Typically, LOCs for NA extraction rely on a structure that can be represented in the simplified scheme of Figure 1 and detailed in the schematized reconstruction of Figure 2: the sample is introduced through an inlet hole; a lysis step inside an inlet chamber or in a dedicated lysis module occurs (whenever not performed by a simple offline procedure); NA are captured into an extraction domain in which washing-elution phases take place, ending with the extract withdrawal through an outlet port (whenever automatic amplification does not occur). Generally, wastes are collected in a specific region of the chip. Sometimes, in case of RNA extraction, there is a dedicated cDNA retrotranscription chamber. Some of these steps can be performed either online (on-chip) or offline (off-chip), depending on aim and structure of the LOC, while others represent the core of the NA extraction process and are performed on-chip.

The choice of the chip structure is a basic step and should be related to the sample type and the extraction protocol, reflecting the overall function of the LOC. Typically, low-complexity samples (e.g., urine, saliva) are processed by chips relying on packed adsorbent matrices, or functionalized surfaces or filtering units, which are designed with a single, long microchannel, filled with the capturing material. In case reagents are pre-loaded on a chip, a single inlet port for sample loading could be sufficient; otherwise, multiple ports should be considered when designing the chip structure. This second design is usually coupled with automatic pump systems for fluids motion, while in the first case a manual syringe could be sufficient. The mixing capacity of the system is a relevant issue to consider when designing a LOC structure, because absence of turbulence (such as in the simplest chips) implies passive diffusion mixing [79]; chips with side channels (forming T junctions) or long coil-shaped microchannels [31] represent optimal strategies to obtain an adequate passive mixing. Coil-shaped channels are also good choices to increase the surface/volume ratio of the adsorbent region, thus increasing the extracted NA quantity. This design also increases the total volume capacity, enabling processing of higher volumes of raw samples.

Typically, chip design includes chambers when extraction protocols need a separated compartmentalization (as in the case of on-chip lysis, or when a strong active mixing is necessary, or when NA are extracted with specific approaches, such as magnetic beads). A design with chambers is also required when successive steps of NA usage are included on a chip (e.g., on-chip amplification). One of the most largely utilized compounds for the fabrication of the main chip structures is PDMS (polydimethylsiloxane), a mineral-organic elastomer particularly useful because it is largely elastically deformable, optically clear, inert, non-toxic, non-flammable and inexpensive. It can be utilized alone, but it is often coupled with harder compounds, like glass or thermoplastic polymers, in layering the main structure of the chip. Because thermoplastic polymers are easily workable and less expensive than glass, several of them are very recurrent: PMMA (polymethylmethacrylate) is the most used alone [26,54,61], but more often it is coupled with PDMS [29,42,45,46,51,60,73]; PC (polycarbonate) was utilized, for example, by Ritzi-Lehnert et al. [71], who developed a PC cassette to be inserted into chip analyzers, by Hwang et al. [68], who developed a microfluidic device composed of four layers of PC, PDMS and glass, and by other authors [52,66]. Although less common, also PET (polyethylene terephthalate) and PS (polystyrene) have been used [50,62]. A good overview of the materials utilized in microfluidic chip fabrication is provided by Ren, Zhou and Wu [80]. A material is preferable to others for its cost-effective availability, optical properties, hardness or capacity of deformability, but it must also assure compatibility with the chemicals used in the protocol. For example, PDMS is not compatible with organic solvents; therefore, it should be excluded when projecting a LOC for extractions requiring such reagents. A quite complete list of chemical compatibilities has been provided by Ali et al. [7]. Advances and automation in manufacturing chips allow the bonding together of the same or different polymers into multi-layers as well as the creation of ports, chambers, channels, filters, sites for the addition of modules, etc. Even the smallest components of the fluidic system (valves, micropumps, micromixers, etc.) can be made of such materials. Some examples are shown with the chips described by Chen et al. [42] and Hwang et al. [68]: in the first study, they etched a part of the main silicon microchannel producing micropillars with the function of a weir-type filtration barrier to retain white blood cells and discard unwanted blood components; in the second study they used a flexible PDMS membrane capable of varying the volume of a microchamber upon occurrence.

While microchannels make up the circuit that transports the sample, the flux within LOCs is enabled and modulated by a motion system. In most of the cases (see Table 1), pressure-driven motion is implemented, where flux is usually sustained by external pumps (syringe pumps or pressure-driven micropumps). Depending on the specific microdevice, they allow sample or reagents circulation at an appropriate, controlled speed. Typically, the starting raw or lysed sample is pumped into the chip at a flow rate ranging between 3 and 7 µL/min, allowing NA to come in contact with the capture matrix. Most of the devices maintain constant flow rate during the whole process, while others modulate access of diverse reagents at different flow rates [9,42,45,52,67,72,77]. Shaw et al. [65] use an electro-osmotic pump operated by an external electric field (100 V/cm) through carbon electrodes positioned upon the inlet holes of each microchannel. Han et al. [26] implement a microfluidic system based on spiral micromixers generated by the operator when pushing different reagents reservoirs. This produces transverse vortex phenomena that self-mix flowing reagents. A first reservoir contains a mix of a lysis and a DNA binding buffer; a second reservoir contains PBS for washing and purification steps; a third contains the elution buffer. Also Jin et al. [58] take advantage of the microvortices generation. They developed a powerless platform with a microchannel that connects thirty-five microwells in series, where the continuous change of cross-sectional area of the flow is able to guarantee an efficient mixing with microvortices.

Another usual approach for enabling motion inside LOCs is represented by magnetic transportation: a movable or a permanent magnet transfers magnetic elements across the microfluidic circuit. Most of the time, magnetic elements are coated with functional groups capable of capturing NA by physical or chemical adsorption and enabling their transfer into collection chambers [30,73]. Sometimes, this strategy is adopted for collecting and removing cell debris [23] or for selectively binding a target (through specific antibodies), for example, for pathogen detection [41]. Magnetic beads appear to be the most versatile and rapid strategy for NA extraction (Table 1). This approach is extremely effective, but inherently more expensive.

**Figure 3:**
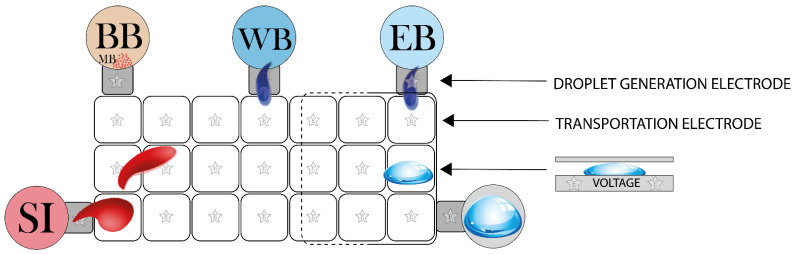
Schematization of a DMF platform. Droplets are formed from the reservoirs (e.g., SI = Sample Inlet, BB = Binding Buffer, WB = Washing Buffer, EB = Elution Buffer) by droplet generation electrodes. Each droplet is controlled in its path by voltage application on all the transportation electrodes.

An interesting scenario where electro-magnetic field is employed is represented by digital microfluidic platforms (DMF), which are based on electrical field modulation for the manipulation of liquid samples in the form of droplets extremely small in size [81,82]. This approach enables the implementation of droplet-based microdevices where, starting from sample, individual droplets are continuously formed and manipulated through the microchannels, or where a variable number of droplets of each reagent are manipulated and continuously moved upon a pathway of transportation electrodes (Figure 3). 

This last case is described in a recent study [22]: electrical and magnetic forces are generated to move and split droplets over the cartridge; the sequence of activation of electrodes and the activation of the magnet represent the automation of this type of microdevices. NA bound to magnetic beads are collected on one side of the droplet, and the separation electrode splits the whole droplet into a minor residual droplet (containing NA which will be eluted) and a major supernatant droplet (containing unbound reagents, which will be washed away). DMF platforms have also been effectively tested for RNA extraction from whole blood [57].

**Figure 4:**
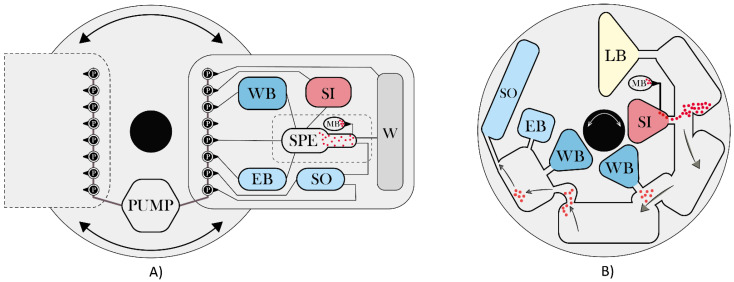
Schematization of (**A**) centrifugal and (**B**) lab-on-a-disk microfluidic systems. Both types, mostly based on a magnetic beads extraction or, less commonly, on silica filtration, are developed onto a rotating platform where sample and reagents motion is enabled by centrifugal and related forces (Coriolis and Euler) that, usually in combination with pneumatic forces, handle the fluidic system and enable sample processing. This principle is applied in traditional rectangular LOCs [36,71,86] and in LOD systems [18,41,51,53,54,60]. These devices generally manage the sample by changing speed and sense of rotation: a particular example of a LOD is represented by LabDisk analyzer [28,64] which implements an automated lyse-bind-wash-elute protocol by means of a gas-phase transition magnetophoresis principle, which combines magnetic and centrifugal forces to carry NA-complexed magnetic beads through the separation liquid/gas interfaces between each reaction chamber. P = pressure application with pump; SI = Sample inlet chamber; SPE = Solid-phase extraction domain; MB = Magnetic beads chamber; LB = Lysis buffer reservoir; WB = Washing buffer reservoir; EB = Elution buffer reservoir; SO = Eluted sample outlet; W = Waste chamber.

Other interesting platforms for NA extraction are represented by centrifugal-driven LOCs (or labs-on-a-disk, LODs), usually coupled with magnetic beads (details in Figure 4), electrokinetic-driven LOCs [32,65], relying on voltage application for motion by electrical gradient, and lab-on-a-paper devices, also implemented for NA extraction [40,55,56]. A recent and cost-effective innovation is microfluidic origami [83]; it is a new branch of microfluidic technology where paper can be exploited as an attractive and inexpensive platform for NA extraction [40,84] due to its intrinsic advantages such as biocompatibility, high surface area, and absorptive nature [85]. 

### 2.2. Raw Samples Pre-Treatment

The first step for a successful NA extraction consists in the effective disruption of the cell. When cells are part of raw tissues (such as stools or solid tissues), the sample needs to undergo a complex pre-treatment (removal of solid impurities, homogenization, etc.) before proceeding with membrane lysis. Membrane lysis methods can be categorized as: (1) mechanical—by grinding, freeze-thawing, potterization, centrifugation, sonication or bead-beating; (2) chemical—by detergents, solubilizing membrane lipids, or by chaotropic agents, perforating cell membranes with the denaturation of trans-membrane proteins, by enzymatic digestion, or by osmotic shock; (3) thermal—heating treatments; or (4) electrical (by membrane dielectric breakdown) [14,87,88]. Sample lysis can be accomplished by a single or a combined use of the above-mentioned methods, according to the type of cell to treat and, eventually, according to the needed quality or yields of the extraction. Specific nucleases can then be utilized to isolate the target NA (to obtain either RNA-free DNA or DNA-free RNA). Finally, removal of contaminants must be performed. A list of proposed approaches for on-chip raw sample pre-processing enabled by diverse LOC strategies is reported in Table 2. Additional details for off-chip pre-processing are reported in Supplementary Materials (Table S1).

Vortexing has been considered comparable to mixing by repetitive pipetting. Serum and plasma separation has been demonstrated to be processable in microfluidic formats and therefore integrable into existing LOCs [64,89,90,91,92]. For this reason, these tissues—although they cannot be defined raw samples—are considered in the present review.

The most used methods for sample pre-processing are chemical, while thermal and mechanical (such as filtering, agitation and pressing) approaches are rarer, and usually applied to lyse bacteria or complex samples such as stools. Common reagents involved in pre-processing include buffers and salts to stabilize pH during lysis, detergents to dissolve cell membranes, and chelating agents which bind to metal ions with two positive charges (e.g., magnesium and calcium) to reduce the level of some enzyme activity such as DNases. When aiming at preserving just the RNA portion after total NA capture, Deoxyribonuclease I (DNase I)—an enzyme able to catalyze the hydrolytic cleavage of phosphodiester linkages in the DNA backbone, inducing DNA degradation—is often added in solution together with lysis buffer [58]. In most on-chip lyses, reagents needed for DNA extraction are administered manually by the operator, before running the process, through side inlet ports or by filling suitable chambers. In advanced architectures, reagents are pre-loaded on-chip. For example, reagents can be encapsulated in low-melting temperature agarose gel and pre-loaded in the respective microchannels and chambers [65], loaded previously, by connecting reagent tubes to the microfluidic system [27], or they can be contained in deformable pouches fabricated into the chip, where depressing the pouch (snap-through) squeezes liquid into the channel [66].

### 2.3. NA Isolation

For NA purification, whose ultimate goal is to remove the interferents from the analyte, producing a solution containing principally the NA portion can be achieved through different approaches [15].

Traditional methods consist of precipitation. In these conventional, widely adopted approaches, cells are lysed, and cell debris is usually removed by centrifugation. In the most used strategy, phase separation is obtained by mixing a solution of organic solvents (typically containing phenol, chloroform and a chaotropic agent, often GuSCN) with the aqueous sample, followed by centrifugation [91,93]. Such reagents are effective in removing proteins, lipids and detergents from the solution, allowing their dissolution or accumulation at the aqueous interface by centrifugation. In particular, GuSCN denatures proteins and RNases; chloroform forms a colorless upper aqueous phase containing RNA, an interphase containing DNA and a lower phenol-chloroform phase containing proteins. NA can be collected from the upper aqueous phase by alcohol (isopropanol or ethanol) precipitation followed by rehydration [7]. Among precipitation methods, salting-out techniques are also often used—based on the exploitation of high concentrations of potassium or ammonium acetate—to allow the removal of contaminants, such as proteins and other biomolecules [15,94]. Although sufficiently effective, these approaches present several drawbacks which make them hard to be implemented on LOCs and POC tests. In fact, they are time-consuming, rely on intensive manual processing, employ hazardous reagents, potentially damaging equipment [95] and, in addition, residual phenol or chloroform may affect downstream applications such as PCR.

Another class of NA extraction methods is liquid–liquid extraction (LLE), based on the manipulation of aqueous pH to extract NA into an organic solvent. Knowledge of the chemical properties of biological specimens allows proper selection of the organic solvent, which is the main factor for successful isolation and purification processes [15,94]. Typical organic solutions and/or detergents for sample washing are phenol, chloroform, or cetyltrimethylammonium bromide. Although widely used for a long time, and best performing on samples with very low NA concentrations, LLE suffers from several disadvantages, which make these methods not easily implementable on LOCs. Limitations include time-consumption, presence of non-automatable steps, usage of organic solvents (which are toxic and costly), and need of expensive glassware. 

#### 2.3.1. Solid-Phase Extraction

More recent approaches overcome drawbacks of precipitation and LLE in on-chip NA extraction. They are called solid-phase extraction (SPE) methods. SPE involves the use of a solid material able to selectively separate the target analyte from the solution, followed by a release mechanism. This approach utilizes chemical and physical properties (i.e., hydrophobic, polar, and/or ionic features) of the solutes dissolved or suspended in a liquid (also known as the mobile phase) to separate them from undesired components when filtered through an adsorbent (also known as the stationary phase). The strong chemistry between the sorbent and the analyte of interest is at the basis of the sorption phase, while weak chemical interactions, such as van der Waals forces (non-polar interactions), dipole–dipole interactions (polar interactions) and hydrogen bonding modulate the retention/release mechanism [7].

SPE in LOCs usually consists of concentrating and purifying analytes from solution by adsorption onto a disposable solid-phase cartridge, followed by elution of the analyte with an appropriate solvent. Adsorption is typically enabled by silica structures, ion-exchange resins, or gels. The most utilized substrate for SPE is represented by silica-based surfaces, which consist of silica material in form of either gel or glass particles, like glass powders, microbeads, microfibers or even microstructures such as micropillars, monoliths or membranes. The principle of silica matrices extraction is based on their exceptional ability in binding DNA under specific salt conditions: modulating pH with a chaotropic binding buffer, silanol groups gain a high affinity to the negative charged backbone of DNA. Moreover, silica material is often covered of positive ions to enhance this affinity. The presence of chaotropic salts at high concentration, coupled to organic solvents (such as ethanol or isopropanol), aids protein denaturation but also facilitates binding of NA to silica structures providing the optimal pH. It is worth to note that chaotropic salts (such as GuSCN and GuHCl) can play a double role in NA extraction, acting both as a lysis agent and being part of the binding buffer. 

**Figure 5:**
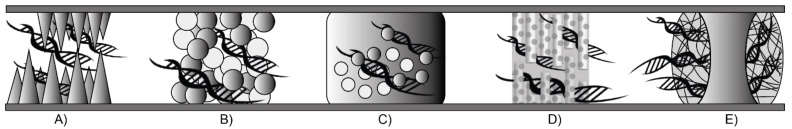
Examples of most common silica structures involved in adsorbing NA: (**A**) pyramidal micropillars [33]; (**B**) surfaces packed with silica microbeads [9,10,38,44,68,76]; (**C**) colloidal sol-gel solutions packed with silica microbeads [24,25,43]; (**D**) silica wafer monolith [31,39,42,65] or polymer monolith with silica microbeads [69,75]; (**E**) silica membrane filters [29,47,53,55,56,59,72].

Different silica structures have been implemented in LOCs for NA extraction (Figure 5). As an interesting example, Brassard et al. [36] utilize a double layer of glass microfiber filters (composed of silica with sodium, potassium or calcium) where the first layer (2.7 µm pored) acts as a pre-filter for whole blood, while the second layer (0.7 µm pored) acts as the capture matrix. Whereas in most cases silica structures are composed of glass, also tetraethyl-orthosilicate (TEOS) [24] and tetramethyl-orthosilicate (TMOS) [38,43] were used as capture materials.

Because chaotropic elements can negatively interfere with downstream processes (such as NA amplification, for which they act as strong inhibitors), alternative SPE methods to a standard silica phase were set up for NA extraction. They consist of using pH dependent anion-exchange approaches [45,96] or kosmotropic salts to induce acidic conditions [97] in order to foster NA binding to the solid phase. An example of a pH-controlled approach is represented by chitosan-coated structures. This strategy promotes NA binding to—and release from—the chitosan phase based on a change in buffer pH. Chitosan is a polymer with a high number of amine groups, which presents a cationic charge at pH 5; but it is easily neutralized at pH 9: this property has been shown to be effective for DNA extraction at pH 5 and its release from chitosan at pH 9 [45]. Moreover, low-molecular weight chitosan is a proven inhibitor of RNases, demonstrating the advantages of chitosan as a solid phase for RNA purification compared to silica [10]. A recent study by Gan et al. [46] showed a chitosan-coated version of the Fusion 5 filter. In this version of the platform, DNA extraction is due to both the entanglement of the long-chain DNA molecules on the filter weave, made of glass fibers bonded with organic binders, and the electrostatic adsorption to the chitosan polymer, which keeps the DNA bound even at high pH (useful feature for successive on-chip amplification, which requires a pH increase). Around pH 5, DNA molecules are “actively” adsorbed onto the chitosan-modified fibers. Once DNA is adsorbed, the physical entanglement of the long-chain molecules with the fiber matrix can also assist the capture. At pH 9, although DNA is not “actively” absorbed onto the fibers, DNA molecules remain bound due to the physical trapping of these long-chain DNA molecules within the fiber matrix against washing and elution.

Another variety of SPE techniques for NA is based on electrostatic or covalent interactions between DNA and surfaces modified through amine coatings. Amine groups below neutral pH have a positive charge (causing negatively charged DNA to bind), which decreases above neutral pH [14]. An example is represented by Shin [52] and Han et al. [26], who use DMA (dimethyl-adipimidate), a non-chaotropic reagent able to bind fragmented DNA to its amino groups, and APTES (3-aminopropyl-triethoxysilane)-coated glass, which captures DNA/DMA complexes. Using the same amino-silanized surface as capture matrix, DMP (dimethyl-pimelimidate) is used as an alternative to DMA, for NA extraction, thanks to its ability of creating complexes with NA by covalent bonds of the amino groups of DMP molecules [58].

Electrostatic interactions were sometimes coupled to nanoporous membrane filtration, obtained for example through mid-sized pores in aluminum oxide membrane [98], to improve NA extraction, as in the case of Kim and Gale [37], who developed a microfluidic system for DNA capture relying on an aluminum oxide membrane with 100 nm pores size. Electrostatic attraction between DNA and alumina causes it to adsorb to the membrane, while other components flow into the waste chamber.

#### 2.3.2. Magnetic Particles

Often, magnetic particles, or beads, have been coupled to suitable buffer systems for rapid and efficient NA extraction. Their use is rapid, simple to perform and can be automated, although more expensive than other methodologies. Magnetic particles work on the principle of complementary hybridization and are composed of a magnetic core, such as magnetite (Fe_3_O_4_) or maghemite (Fe_2_O_3_), usually coated with a matrix of polymers, silica, or hydroxyapatite with terminal functionalized groups to enable NA capture. The most recurrent covalent functionalizations of magnetic beads are carboxy-, amino-, hydroxy-, thio- terminations of the coated surface, while the non-covalent ones are represented by bioactivated coatings (peptides, aptamers, biotin, streptavidin, etc.) or rely on hydrophobic interactions [99]. The method is based on the principle that NA can reversibly bind the solid surface, previously coated with NA binding antibodies, aptamers, or with functional groups that interact specifically with DNA. The reversible complexation of beads with nucleic acids is controlled by adjusting the pH or the salt concentration [100]. For LOC devices, the advantage of using magnetic particles is that they can be used free floating in solution, thereby maximizing the interaction between sample and beads, and then collected in a microchannel or a microchamber using a magnetic field, rather than by centrifugation or filtration.

**Figure 6:**
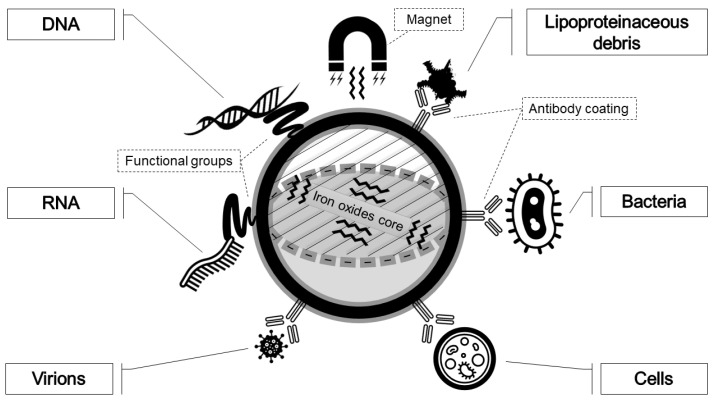
Magnetic beads can be magnetically manipulated because of their iron core and—with their activated coatings, such as functional groups or specific antibodies—can interact and selectively bind a desired ligand. The most recurrent functionalization for NA extraction is the silica-coating. If the target is human mRNA, the easiest choice is magnetic beads coated with oligo-dT probes [48]. When aiming only at a pathogen detection, functionalization with target specific antibodies is used [41], although this falls outside the aims of the review, devoted to total NA purification.

Magnetic beads (Figure 6) represent the most commonly adopted strategy for NA extraction. After binding, beads are separated from contaminating cellular components, washed, and exposed to ethanol to obtain a purified DNA elution [101]. The majority of LOCs based on a magnetic beads NA extraction utilize beads with silica coatings (Table 1); they are added and mixed to sample and lysis buffers [28,49,50,51,54,60,61,63,64,67,70,71,73]. After cell lysis, DNA-bound beads are driven and collected by a magnetic coil, while the unbound debris is removed by a stream of washing buffers. Some approaches couple magnetic field with heating to improve the whole process [27]. For example, Lee et al. [23] describe a compact device based on a laser-irradiated magnetic beads system (LIMBS). It is equipped with carboxylic acid-terminated polystyrene magnetic beads, involved in heat transfer and cell debris adsorption, and operates with a laser-beam irradiation (1 W for 40 s), able to heat magnetic beads. The continuous vibration occurring on the chip by means of a motor is responsible for beads collision with the cellular targets, enabling heat transfer to them. The combination of heat shock and mechanical agitation breaks membranes, making cells release nucleic acids in solution. Functional groups on the polystyrene surface of the magnetic beads have the capacity to adsorb most of PCR inhibitors, like denatured proteins and cell debris, which are removed by capture of beads with a permanent magnet. Another interesting strategy is presented by Lien et al. [35], who exploit a double step of magnetic beads usage in DNA extraction from whole blood: antibody-labeled leukocyte-specific magnetic beads, for enabling leukocytes separation, and a second type of magnetic beads (charge-switchable), able to switch charge by pH variations to bind/unbind DNA molecules. 

#### 2.3.3. Other Approaches

Although rarely, other materials different from silica nano- or micro-structures or magnetic-based components were used for SPE. As capture matrix, Huang et al. [74] utilize a porous polymer monolith SPE-column composed of ethylene dimethacrylate (EDMA, as crosslinker), butyl-methacrylate (BUMA), 1-dodecanol (as porogenic solvent), azobisisobutyro-nitrile (AIBN, as polymerization initiator). Differently from traditional SPEs—which exploit particle-based adsorbents—polymer monoliths, which are solid pieces of stationary phase, present bimodal structures: macropores enable passage of solvent while mesopores separate analytes. These methods assure separation capabilities similar to those of particle-based adsorbents, increasing sample throughput, reducing solvent usage, and being suitable for automation [102]. Another example is described by Choi et al. [62] who present the functional coating of a polycarbonate chip with an adsorbing surface made of a DMAMS (poly-2-dimethylaminomethyl-styrene) polymer. Such material is positively charged, so it has the capacity to electrostatically attract the negative phosphate backbone of DNA. Finally, strategies different from SPE were also adopted to extract DNA on LOCs, for example those relying on isotachophoresis [32].

### 2.4. Additional Considerations on RNA Isolation

Specific considerations can be associated to RNA extraction. When working with RNA, chemical differences with DNA must be considered. In fact, RNA is very different from DNA in terms of chemical stability. The single –OH group difference makes the ribose sugar much more chemically reactive than deoxyribose. Additionally, RNases, a class of RNA degrading enzymes ubiquitous and robust, can rapidly destroy RNA molecules. Due to RNA fragility, a reverse transcription phase to complementary DNA (cDNA) is necessary in order to stabilize molecules and allow further processing after extraction.

RNA is more closely related to proteins than DNA, so it is able to describe a scenario tightly connected to the actual cellular machinery. Nevertheless, whereas a large number of LOCs were developed for DNA extraction, microfluidic systems dedicated to RNA purification are relatively rare in literature. This is probably due to its high instability and susceptibility to degradation, thus requiring special care and precaution during isolation and processing. Moreover, due to affinity of many matrices (such as the silica ones) for both DNA and RNA, most of the extraction systems finely tuned for DNA also bind RNA, thus reducing the need for RNA dedicated solutions.

In these systems, both NA types are isolated from the same biological sample at the same time, and extraction specificity is usually assured by the endonuclease activity of either DNase I or RNase A modulating their action to recover either RNA or DNA, respectively. Strong denaturants are typically used in intact RNA isolation to inhibit endogenous RNases, which are heat-stable and refold following heat denaturation [11].

Reedy et al. [9] perform both DNA and RNA extractions with their LOC, and, when only RNA was needed, DNA was digested at the end of the extraction process. Also Hagan et al. [10] digest DNA at the end of the extraction with a DNase. Nevertheless, in some cases, specific protocols were implemented for RNA purification. Capture of eukaryotic mRNA, which is clearly characterized by a poly-A tail (a sequence of polyadenylic acid at the 3′ terminus), has been performed exploiting magnetic beads coated with oligo-dTs. Additionally, Han et al. [14], who perform mRNA extraction and cDNA synthesis on the same device, utilize oligo-dT magnetic beads, which here are led to the elution chamber by taking advantage of lateral magnetophoresis technique. Another example of poly-T functionalization is described by Lee et al. [48] in their lateral magnetophoresis mRNA microextractor, and also by Satterfield et al. [103] with the surface of a methacrylate-based porous polymer monolith (PPM) functionalized with 20-mer oligo-dT.

When the chip included a PCR module or the RNA target was immediately amplified by off-chip PCR, as it is common for viral RNA detection in human specimens, generally both nucleic acids are extracted together with no need of endonuclease digestion.

### 2.5. Post-Processing

NA purification on LOCs can be achieved using either of the two following strategies: selectively binding NA to the adsorbent matrices while washing out, usually with a nonpolar solvent, the rest of cell components and other chemicals, and further eluting NA with appropriate solvent for recovery; or not retaining NA, while capturing all components to discard [23]. The first approach is the most exploited.

A washing buffer is used to remove non-nucleic acid organic (i.e., proteins, lipids) and inorganic molecules (i.e., unbound components, residual detergents, lysing salts) from resultant extracts. This phase usually consists of washing the NA-adsorbed surface with a slow, controlled flow of propanol, generally applied for 15 min, and in collecting wastes into a dedicated chamber or through an outlet port. Sometimes ethanol or ethanol-based buffers are preferred to propanol (Table 3). In some LOCs, there is a unique washing step; in other cases, there is a pre-wash phase with alcoholic mixed salt buffers and a main washing step with an alcoholic buffer. Less commonly, in some specific cases with magnetic beads [73], samples can be washed by forcing their passage through an immiscible washing oil phase capable of blocking molecules to discard. In some implementations, protein separation is performed before NA capturing, using protein-capture columns [38].

Analogously to the other chemicals and independently of the chosen washing strategy, in most cases, the washing buffer is injected by syringe pumps or is pre-stored on-chip in dedicated chambers, which are activated by pressure application or by valve opening at the correct time during the purification protocol. In some implementations, vice versa—the sample is moved into the chamber containing the washing buffer. As a consequence of washing, lysed biomolecules (such as proteins, metabolites, membrane lipids, etc.), lysing detergents and any buffers used to adjust pH conditions are removed, and NA molecules remain bound to the adsorbent substrate. To be released from the capture matrix, NA must come in contact with a low ionic strength buffer, whose pH is adequate to dissolve the bonding. The elution buffer collects NA and leads it to the desired region, to gather it or for subsequent on-chip detection aims. 

Elution strictly depends on the pH of the solution, which enables or disables binding, and it also directly depends on the used capture matrix. DNA elution from silica matrix is performed by a hypo-osmotic solution, typically a low salt buffer or elution buffer, generally alkaline. A common NA elution consists in flowing pH = 8 TE buffer (Tris-EDTA). Chitosan has a cationic charge which is easily neutralized at pH = 8.5/9; therefore, NA elution from this matrix is commonly obtained by using pH = 9 TrisKCl [9,45]; the same buffer is used when eluting from aluminum oxide membranes because it showed better extraction efficiencies [37]. The same buffers are used for elution from silica-coated magnetic beads. Other alkaline eluents are utilized, for example NaOH or NaHCO_3_, but, even though a higher pH yields a more concentrated extraction, water is always widely used. In sample-in answer-out devices, where an amplification occurs after NA extraction, elution is performed directly into the PCR mix. Elution flow rates can vary significantly, depending on the microchannel section and the matrix composition, from a higher rate of 500 µL/min [68] to a slower rate of 1 µL/min [45]. It is worth to note that the elution volume represents a critical issue; in fact, while volume reduction is an advantage in terms of costs and wastes reduction, it can be troublesome for pipetting away small NA quantities in minimal elution volumes. To overcome this issue, an over-dilution step of the final eluate could be considered (e.g., Lee et al., 2020 [49]), or parallel extractions from the same raw sample should be enabled when designing the chip.

## 3. NA Applications for Medical Diagnostics

As final validation, many LOCs are tested for the application in different molecular diagnostic fields to clarify patients’ genetic anomalies or occurring infections caused by pathogens, such as bacteria, DNA viruses, parasites, etc. The extraction step is crucially important because NA quality influences the success of further analyses. Many LOCs have been coupled to DNA detection methods, usually performing both steps online, relying on amplification to identify the unique sequences of specific invading pathogens within human samples. Rapid and comfortable (possibly run ‘at the time and place of patient care’) identification of infectious diseases can reduce further treatment costs and patient sufferings, and can contain disease spreading among the population. LOCs were fruitfully applied to detect many different pathogens from different raw sample types (Table 4).

Although more often used for detection of proteins, metabolites or other small molecules, extraction LOCs can also be helpful in genetic disease detection [104], usually performed through DNA offline extraction followed by sequencing or genotyping approaches. More recently, LOCs were designed to capture circulating cell-free DNA (ccfDNA) from serum samples of cancer patients [105,106,107]: ccfDNA capture requires increased ability in adsorbing short fragments, thus implying customized isolation strategies [19]. Another interesting application of DNA extracted with LOCs is represented by forensic evaluations, especially when available sample volumes are extremely low [9,24,29,75]. While DNA extraction represents the basis for analyses regarding human genotype and for most of the infections caused by pathogens, RNA extraction enables procedures to shed light on the phenotypic layer of the organism, interesting to identify either pathological or specific physiological conditions detectable at the transcription level. Moreover, RNA extraction was exploited on LOCs for the detection of RNA viruses infecting human organism (Table 4).

The recent COVID-19 pandemic has increased the need for rapid diagnoses to isolate infected population and, moreover, the need for rapid and easy-to-use devices for an effective screening of population and contact tracing of infected patients. This scenario has meant that the world of miniaturized devices was prepared to offer its own alternatives to the labor-intensive traditional RT-qPCR. A not-exhaustive list of already-validated LOCs for SARS-CoV-2 detection [108] is presented in Table 5. Generally, these devices, considered more as POC tests, rely on total NA extraction followed by RT-qPCR, performed on pre-filled cartridges or cartridges to fill with a kit of reagents for a complete processing.

## 4. Conclusions

The present review aims at describing the strategies adopted in the literature to extract NA from raw human samples. Most LOCs were developed for genomic DNA extraction only, generally addressed to a subsequent off-device PCR analysis with bench thermocyclers, although RNA or total NA extraction are also described, often coupled with a detection domain. Diverse microfluidic solutions have been analyzed, differing in the starting raw sample, the shapes of the fluidic path, the motion strategy of analytes and reagents, the adsorbent structures, the methods for eluate and wastes collection, or the further on-chip detection. For each phase, LOC developers have to choose among a plethora of possible solutions, potentially considering intrinsic issues to take care. For example, the adsorption capacity of the chemical composition of the chip should be considered to avoid non-specific capture. A quite common case is represented by PDMS channels, which can adsorb biomolecules onto the walls [80]. To avoid this, several strategies have been proposed, such as plasma treatment, surface coating with organosilanes, or other types of functionalization. Nevertheless, most of the analyzed LOCs demonstrated extraction efficiencies similar to commercial kits, thus demonstrating that these kinds of drawbacks are relevant only when dealing with very little concentrated targets (e.g., ccfDNA, rare RNA transcripts, or low bacterial/viral presence).

The extraction strategies most compatible with LOC miniaturization are SPE methods relying on silica adsorbent matrices of various shape, layering, chemical composition and filtering capacity. SPE approaches are very efficient, although they need high volumes of reagents to bind, wash and efficiently elute. NA. Moreover, the release of shorter fragments during the elution phase could represent a difficult step when using silica matrices [7,50]. The obstruction of the extraction matrix, especially when whole blood is used, should be avoided by an effective pre-treatment of the sample or by specific flow control strategies (e.g., crossflow filtration [42]). Silica matrices are often chosen as a capture method for NA because they are easy to be integrated in miniaturized instruments, provide a high-purity eluate, and are less expensive than other matrices. Conversely, magnetic beads are the adsorbent matrix of choice in many other cases, especially in LODs, for their ease of usage, high flow rate during the extraction compared to silica matrices [16], and because they are versatile and can be coated with generic or specific functionalized layers to optimize capture precision. Nevertheless, they represent an expensive solution and often require a bulky electrical device or a unit capable of modulating magnetic field. Moreover, magnetic beads tend to aggregate, a problem that can be avoided not exceeding in magnetic force application or in motion/centrifugal speed.

LOCs for NA extraction are usually tested with whole blood samples because, on the one hand, it is a challenging sample for the development of effective capture matrices, but, on the other one, it is quite common and obtainable, commonly used for genomic DNA extraction. Other kinds of samples, such as nasal washes, aspirates, swabs or stool are mostly processed for total NA extraction in LOCs devoted to pathogens detection. Ideally, it would be possible to use the same LOC to obtain NA from various types of raw samples, adapting the pre-treatment step to adequately free NA in solution. It is therefore of paramount importance to adopt lysis protocols suitable for the specific sample.

Typically, a nucleic acid extraction performed with traditional kits and laboratory equipment can last from about 20 min to 1–3 h [117,118,119,120,121], and expert operators and expensive facilities are required. Once optimized for accuracy, miniaturization, versatility and automation, LOCs will overcome the disadvantages of traditional approaches. Indeed, the main objective of LOCs is to obtain reliable results while dramatically simplifying the entire process and reducing running time and needed volumes. These aspects would be particularly appealing, for example, in the forensic context, where the amount of available samples is often limited. Moreover, LOCs allow one to maximize experimental design, overcoming the limiting factors related to running time and reagents consumption. Finally, thanks to their compactness, portability and user-friendliness, LOCs allow the performance of on-field analyses; instead of moving samples to the laboratory, it would be possible to move the laboratory equipment to end-users, with a resulting significant reduction of sample contamination and degradation. In this perspective, LOCs can foster the development of POC tests, promoting and supporting personalized medicine, wide population screening and telemedicine.

## Figures and Tables

**Table 1:** Summary of the performances for considered LOCs. Type and volume of starting biological sample, on- or off-chip lysis, NA extraction yield, principles, and total time (including pre-processing, when declared).

Sample Type	Sample Volume	Process Time	Lysis	NA Yield/Limits of Detection (in Eluted Volume)	Extraction Matrix and Motion Principles	Ref
Whole blood	200 µL	30 min	On	Total NA, ∼10^5^ DNA copies (in 100 µL)	Silica-coated magnetic beads, magnetic and centrifugal-driven	[28]
Whole blood	1 µL	7–8 min	On	Genomic DNA, 21.8 ± 2.3 ng/µL	Glass fiber filter, pressure-driven	[29]
Whole blood	0.1–100 µL	1 min	Off	Human mRNA Yield n.a.	Oligo-dT magnetic beads, lateral magnetophoresis	[30]
Whole blood	10 µL	<20 min	On	Genomic DNA, 39.7 ng/µL (in 60 µL)	Porous silicon wafer, pressure-driven	[31]
Whole blood	1 µL	n.a.	On	Pathogen DNA Yield n.a.	Isotachophoresis, electrokinetic-driven	[32]
Whole blood	50 µL	<60 min	Off	Genomic DNA Yield n.a.	Silica micropillars, pressure-driven	[33]
Whole blood	5.4 µL	<30 min	On	Pathogen DNA, ∼30 ng/µL (in 25 µL)	Magnetic beads, magnetic-driven	[34]
Whole blood	200 µL	20 min	On	Genomic DNA, 33.26 ± 2.5 ng/µL (in 5 µL)	Antibody-coated magnetic beads, pressure-driven	[35]
Whole blood	300 µL	27 min	On	Genomic DNA, 54.3 ng/µL (in 200 µL)	Glass fiber filter, centrifugal-driven	[36]
Whole blood	20 µL	<10 min	Off	Genomic DNA, 38.8 ng/µL (in 25 µL)	Aluminium oxide membrane, pressure-driven	[37]
Whole blood	10 µL	n.a.	Off	Genomic DNA, 24 ± 0.2 ng/µL (in 2 µL)	Surface packed with silica beads + TMOS monolith, pressure-driven	[38]
Whole blood	1 µL	n.a.	On	Genomic DNA, 10 ng/µL (in 70 µL)	Silica monolith, pressure-driven	[39]
Whole blood	100 µL	23 min	Off	Pathogen RNA, LoD 10^3^–10^6^ copies/mL (in 100 µL)	Glass fiber filter, capillary absorption	[40]
Whole blood	100 µL	12 min	On	Pathogen DNA Yield n.a.	Antibody-coated magnetic beads, magnetic and centrifugal-driven	[41]
Whole blood	0.09 µL	n.a.	On	Genomic DNA Yield n.a.	Magnetic beads, electrokinetic-driven	[22]
Whole blood	5 µL	50 min	On	Genomic DNA, 35.7 ng/µL (in 35 µL)	Porous silicon wafer, pressure-driven	[42]
Whole blood	4 µL	9–30 min	Off	Genomic DNA Yield n.a.	Sol-gel packed with silica beads, pressure-driven	[25]
Whole blood	10 µL	<30 min	Off	Genomic DNA, ∼9.8 ng (in 70 µL)	Sol-gel packed with silica beads, pressure-driven	[43]
Whole blood	4 µL	<10 min	Off	Total DNA Yield n.a.	Surface packed with silica beads, pressure-driven	[44]
Whole blood	5 µL	10 min	Off	Genomic DNA, 48.7 ng (in 28 µL)	Chitosan-coated surface, pressure-driven	[45]
Whole blood	0.5 µL	20 min	On	Genomic DNA, 19.3 ± 4.9 ng/µL (in 100 µL)	Chitosan-coated glass fiber filter, pressure-driven	[46]
Whole blood	n.a.	<5 min	Off	Total RNA Yield n.a.	Glass fiber filter, pressure-driven	[47]
Whole blood	50 µL	1 min	Off	Human mRNA Yield n.a.	Oligo-dT magnetic beads, magnetic-driven	[48]
Whole blood	1 µL	40 min	Off	Genomic DNA, 8 ng/µL	Magnetic beads, magnetic-driven	[49]
Whole blood	5 µL	5 min	On	Pathogen NA, 16–35 ng/µL (in 20 µL)	Magnetic beads, magnetic-driven	[50]
Whole blood	4 mL	<15 min	On	cfDNA Yield n.a.	Silica-coated magnetic beads, centrifugal-driven	[51]
Whole blood	2 µL	n.a.	On	Genomic DNA Yield n.a.	DMA and APTES-coated surface, pressure-driven	[52]
Whole blood	10 µL	n.a.	On	Pathogen DNA, LoD 100 CFU/mL	Glass fiber filter, centrifugal-driven	[53]
Whole blood	500 µL	15 min	On	Pathogen DNA, 100 copies/mL	Magnetic beads, magnetic and centrifugal-driven	[54]
Whole blood	30 µL	2 min	On	Pathogen DNA, 10.000 copies/mL	Glass fiber filter, capillary absorption	[55]
Whole blood	20 µL	3 min	On	Pathogen DNA Yield n.a.	Glass fiber filter, capillary absorption	[56]
Whole blood	25–100 µL	20 min	Off	Total RNA, ∼250–450 ng (in 10 µL)	Magnetic beads, electrokinetic-driven	[57]
Plasma	140 µL	<30 min	On	Total NA, ∼40 ng/µL (in 20 µL)	DMP and APTES-coated surface, pressure-driven	[58]
Plasma	500 µL	15 min	/	cfDNA, 10–100 ng/µL	Glass fiber filter, pressure-driven	[59]
Serum	30 µL	2 min	On	Pathogen DNA, 10.000 copies/mL	Glass fiber filter, capillary absorption	[55]
Serum	200 µL	<10 min	On	Pathogen DNA, LoD 8 copies/reaction	Silica-coated magnetic beads, centrifugal-driven	[60]
Serum	50 µL	1 min	On	Pathogen NA, LoD 1000 copies/mL	Magnetic beads, magnetic and pressure-driven	[61]
Serum	n.a.	30 min	Off	Pathogen DNA Yield n.a.	Poly-DMAMS-coated surface, chemical adsorption	[62]
Serum	100 µL	n.a.	On	Pathogen DNA Yield n.a.	Magnetic beads, automatic pipetting	[63]
Serum	200 µL	30 min	On	Pathogen DNA Yield n.a.	Silica-coated magnetic beads, magnetic and centrifugal-driven	[64]
Serum	0.4 µL	<1 min	On	Pathogen DNA Yield n.a.	Carboxyl magnetic beads, magnetic-driven	[23]
Buccal sample	500 µL	7–8 min	On	Genomic DNA Yield n.a.	Glass fiber filter, pressure-driven	[29]
Buccal sample	20 µL	n.a.	On	Genomic DNA, 4.03 ± 0.6 ng/µL	Silica-based monolith, electrokinetic-driven	[65]
Buccal sample	100 µL	n.a.	On	Pathogen NA, LoD 10^3^ cells-virions/mL	Glass fiber filter, pressure-driven	[66]
Buccal sample	100 µL	10 min	On	Genomic DNA, 50.45 ng/µL (in 125 µL)	Silica-coated magnetic beads, pressure-driven	[67]
Buccal sample	10 µL	9–30 min	Off	Genomic DNA Yield n.a.	Sol-gel packed with silica beads, pressure-driven	[25]
Buccal sample	20 µL	3 min	On	Pathogen DNA Yield n.a.	Glass fiber filter, capillary absorption	[56]
Buccal sample	100 µL	n.a.	On	Pathogen DNA Yield n.a.	Magnetic beads, automatic pipetting	[63]
Nasal sample	30 µL	2 min	On	Pathogen DNA, 10.000 copies/mL	Glass fiber filter, capillary absorption	[55]
Nasal sample	50 µL	n.a.	On	Pathogen DNA, LoD 1000 CFU/mL	Glass fiber filter, centrifugal-driven	[53]
Nasal sample	1 mL	<20 min	On	Pathogen DNA, LoD ∼61 CFU/mL	Surface packed with silica beads, pressure-driven	[68]
Nasal sample	100 µL	n.a.	Off	Pathogen RNA, LoD 10^3^ copies/mL	Silica-based monolith, pressure-driven	[69]
Nasal sample	30 µL	15 min	Off	Pathogen RNA Yield n.a.	Silica-coated magnetic beads, powerless magnetic-driven	[70]
Nasal sample	500 µL	n.a.	On	Pathogen RNA Yield n.a.	Silica-coated magnetic beads, magnetic and centrifugal-driven	[71]
Nasal sample	8 µL	<10 min	Off	Pathogen DNA Yield n.a.	Surface packed with silica beads, pressure-driven	[44]
Nasal sample	10 µL	9–30 min	Off	Pathogen DNA Yield n.a.	Sol-gel packed with silica beads, pressure-driven	[25]
Nasal sample	100 µL	n.a.	On	Pathogen NA Yield n.a.	Glass fiber filter, pressure-driven	[72]
Stools	200 mg	<40 min	On	Pathogen NA, 46.4 ng/µL of RNA (in 200 µL) 68.4 ng/µL of DNA (in 200 µL)	Magnetic beads, pressure-driven	[27]
Stools	400 µL	7 min	On	Pathogen DNA, 59.3 ng/µL (in 10 µL)	Silica-coated magnetic particles, electromagnetic-driven	[73]
Stools	180–220 mg	45 min	Off	Pathogen DNA, LoD 0.125 pg	Porous polymer monolith, pressure-driven	[74]
Urine	50 µL	10 min	On	Genomic DNA Yield n.a.	DMA and APTES-coated surface, pressure-driven	[26]
Urine	10 µL	n.a.	On	Genomic DNA Yield n.a.	DMA and APTES-coated surface, pressure-driven	[52]
Urine	20 µL	3 min	On	Pathogen DNA Yield n.a.	Glass fiber filter, capillary absorption	[56]
Urine	100 µL	<40 min	On	Pathogen DNA, LoD∼10 CFU/mL	Porous polymer monolith packed with silica beads, pressure-driven	[75]
Semen	2 µL	n.a.	On	Genomic NA Yield n.a.	Surface packed with beads (silica + chitosan-coated silica), pressure-driven	[9]
Semen	4 µL	16 min	Off	Total RNA Yield n.a.	Surface packed with silica beads, pressure-driven	[10]
Semen	1 µL	n.a.	On	Genomic DNA Yield n.a.	Sol-gel packed with silica beads, pressure-driven	[24]
Semen	5 µL	9–30 min	Off	Genomic DNA Yield n.a.	Sol-gel packed with silica beads, pressure-driven	[25]
Semen	1.5 µL	17 min	Off	Genomic DNA, 17 ng (in 15 µL)	Surface packed with silica beads, pressure-driven	[76]
Spinal Fluid	40 µL	<30 min	Off	Genomic DNA Yield n.a.	Sol-gel packed with silica beads, pressure-driven	[43]

**Table 2:** Pre-treatment procedures applied to different sample types and in diverse LOC strategies. In details: guanidinium salts, principally GuHCl (Guanidine hydrochloride) and GuSCN (Guanidine thiocyanate), are the most used chaotropic agents with the capacity of solubilizing proteins. Octylphenolethoxylate, commonly known as Triton-X100, is a surfactant used to extract and release the cellular content due to its reactivity with lipid membrane bilayer. DDT (Dithiothreitol) is primarily used to reduce disulphide bonds and, for example, deprotect thiolated DNA (sperm cells) or denature protein structures. Proteinase K is an enzyme used to digest protein contaminants and inactivate nucleases. Tris (tris(hydroxymethyl)aminomethane) is able to maintain pH of the buffer at a stable point, usually 8.0, and to interact with lipopolysaccharides of the membrane, destabilizing it; EDTA (ethylenediaminetetraacetic acid) is a chelating agent which binds to metal ions (e.g., magnesium and calcium), making them unavailable for other reactions such as those related to DNases activity, or breaking Gram-negative bacteria walls. SDS (sodium dodecyl sulphate) is a protein denaturing detergent. CTAB (cetyltrimethylammonium bromide) solubilizes membrane lipids and promotes cell lysis. Lysozyme is an antimicrobial enzyme able to break thick peptidoglycan structures and lyse Gram-positive bacteria, useful when aiming at infection detections. NaOH is used to perform alkaline lysis of membranes by the saponification of lipids. RNases catalyze RNA degradation. SV = Sample volume; BV = Buffer volume.

ON-CHIP SAMPLE PREPARATION					
**CHEMICAL LYSIS**					
**Sample Type**	**SV**	**BV**	**Reagents**		**Ref**.
Whole blood	90 nL	n.a.	Sample droplet electrokinetically moved, mixed in a loop motion and incubated with Proteinase K and lysis buffer (details n.a.)		[22]
	1 µL	100 µL	Sample directly pipetted onto the extraction membrane. Water (200 µL) is aspirated over the sample and the membrane by the syringe pump. 10 mM NaOH (100 µL) is loaded into the extraction chamber and incubated for 5 min. 1 mM HCl (50 µL) is loaded to neutralize pH after lysis		[29]
	10 µL	50 µL	Sample and lysis buffer (4 M GuSCN in TE; 1% Triton X-100; pH 6.7) simultaneously pumped from two inlet holes into the microchannel		[31]
	200 µL	n.a.	Sample mixed with lysis buffer, RNase-A, binding buffer (details n.a.)		[35]
	0.5 µL	50 µL	Sample mixed into the reaction chamber with a lysis buffer solution (0.1% CTAB, 1.5 M NaCl, MES, pH 5.0) and incubated for 15 min at room temperature		[46]
	4 µL	5.6 µL	Sample is diluted with PBS (if the blood is old), mixed with lysis buffer (5.6 µL), and incubated for 5 min		[49]
Plasma	140 µL	500 µL	Sample, DMP, lysis buffer (100 nM TrisHCl pH 8, 10 nM EDTA, 1% SDS, 10% Triton X-100), Proteinase K, DNase I (in case of RNA extraction) incubated for 20 min		[58]
Saliva	100 µL	200 µL	Sample mixed with the cell lysis solution (Proteinase K and GuSCN-based lysis buffer)		[67]
	100 µL	100 µL	Sample mixed with pre-stored lysis and binding buffer (GuHCl-based)		[68]
	500 µL	100 µL	Sample pipetted into loading chamber. Water (200 µL) is aspirated over the sample and the membrane by the syringe pump. 10mM NaOH (100 µL) is loaded into the extraction chamber and incubated for 5 min. 1 mM HCl (50 µL) is loaded to neutralize pH after lysis		[29]
Buccal swab	100 µL		Swab is placed into loading chamber. Water (200 µL) is aspirated over the sample and the membrane by the syringe pump. 10 mM NaOH (100 µL) is loaded into the extraction chamber and incubated for 5 min. 1 mM HCl (50 µL) is loaded to neutralize pH after lysis		
	20 µL		Swab incubated into the lysis solution (5 M GuHCl in 10 mM TE) at room temperature for 10 min		[65]
Semen	2 µL 20 µL	480 µL 496 µL	Lysis for DNA extraction: semen (2 µL) diluted 1:1 with water, mixed with lysis buffer (496 µL, 6 M GuHCl with 40 mM DDT, pH 6.1) Lysis for RNA extraction: neat semen (20 µL) mixed with lysis buffer (480 µL, 6 M GuHCl with 40 mM DDT, pH 6.1)		[9]
	1 µL	n.a.	Sample loaded and mixed with lysis buffer (6 M GuHCl with 4 mM DDT)		[24]
Urine	50 µL	155 µL	Sample mixed with a lysis-binding solution containing a GuHCl-based lysis buffer (AL buffer, Qiagen), Proteinase K and DMA binding agent		[26]
Stool	400 µL		Liquid stool (diluted in water) mixed with pre-charged guanidine solid salts (reconstituting to 5 M GuHCl) and incubated for 5 min		[73]
**MECHANICAL + CHEMICAL LYSIS**					
**Sample type**	**SV**	**BV**	**Mechanical step**	**Reagents**	**Ref.**
Hematuria urine	100 µL	100 µL	Solution is pressure-forced (150 psi) through small pores polymer monolith	Sample is mixed with lysis solution containing Proteinase K (0.8 mg/mL), GuSCN and SDS (0.01%)	[75]
Whole blood, Urine	2–10 µL	n.a.	Filtration	Sample is mixed with Proteinase K and AL Buffer, Qiagen	[52]
Whole blood	1 µL	n.a.	Sample is premixed with PBS by the micromixer to adjust viscosity of the circulating solution. Sample flows through a pillar filtering structure of 3 µm spacing, which retains white blood cells (6–9 µm diameter) and lets other blood components pass through	Collected white blood cells are mixed with 6 M GuHCl	[39]
Nasal swab	1 mL		Swab is priorly vortexed for 1 min to detach cells. Solution is loaded and the vibrating and flexible PDMS valve makes beads collide and cells to be captured, while providing a strong mixing	NaOH (6 µL; 0.02 N) is loaded for lysis	[68]
Whole blood	5 µL	150 µL	Filtration with 3.5 µm pores structure to retain white blood cells and discard plasma and red blood cells	Blood in 0.9% NaCl (45 µL). Mixing white blood cells with loading buffer (1% Triton X-100 and 6 M GuSCN, pH 6.4)	[42]
Nasopharyngeal swab	100 µL	600 µL	Mixing occurs by air bubble insufflation	Sample is mixed with pre-treatment buffer (300 µL; PBS and Lysozyme) and with a GuSCN-based lysis buffer (300 µL). Incubated at room temperature for 3 min	[72]
Whole blood, serum, plasma	200 µL	600 µL300 µL	Constant mixing is provided by the control of rotational frequency	Sample is loaded with a lysis solution (600 µL; GuSCN or AL Buffer, Qiagen, Triton X-100, EDTA) or a GuHCl-based lysis buffer (300 µL; AL Buffer, Qiagen). Sample is incubated with lysis solutions for 15 min at room temperature	[28,64]
**MECHANICAL + THERMAL LYSIS**					
**Sample type**	**SV**		**Mechanical step**	**Thermal step**	**Ref.**
Serum	0.4 µL		Agitation	Irradiation (40 s) of the vibrating chamber with laser beam (808 nm) for heat shock	[23]
**CHEMICAL + THERMAL LYSIS**					
**Sample type**	**SV**	**BV**	**Reagents**	**Thermal step**	**Ref.**
Nasopharyngeal swab	500 µL	500 µL	Sample is mixed with lysis buffer (500 µL; ML Buffer, Qiagen) and Proteinase K	Lysis chamber is heated with a resistive heater	[71]
Whole blood	1 µL	14 µL	Sample is mixed with lysis-electrolytic buffer (13 µL; 50 mM Tris, pH 8.2; 50 mN HEPES, 1 µL of Proteinase K)	Heating for 3 min with an on-chip resistive heater	[32]
**THERMAL + MECHANICAL + CHEMICAL LYSIS**					
**Sample type**	**SV**	**BV**	**Thermal and mechanical steps**	**Reagents**	**Ref.**
Whole blood	5.4 µL	5.8 µL	Chip heated and agitated by a magnet (56 °C for 6 min)	Sample is mixed with lysis buffer (5.4 µL, details n.a.) and Proteinase K (0.4 µL).	[34]
Whole blood	300 µL	330 µL	Heating with a thermoelectric heater (56 °C for 10 min). Mixing with an air bubble blow from the bottom of the chamber	Sample is mixed with Proteinase K (30 µL) and lysis buffer (300 µL; AL Buffer, Qiagen)	[36]
Stool	200 mg	n.a.	Sample is heated (90 °C for 5 min) and homogenized by a small vibrating magnet. Homogenate mixed by air bubble insufflation and filtered by a 1 µm pore-size filter to remove fecal impurities	Lysis reagents (details n.a.) are mixed with sample by air pressure application.	[27]

**Table 3:** Most used reagents for washing and elution steps in LOCs.

**Washing Buffers**	**Reference**
Propanol	[24,25,33,37,38,43,44,56,76]
Ethanol	[10,31,40,42,52,59,66,69,74,75]
Ethanol-based	[22,27,28,36,53,64,65,71]
PBS	[26,41,58]
Others	Tris-HCl [67]; GuHCl [50,73]; MES [9,45]; SDS [46]; NaOH [55]; Water [62]
**Elution Buffers**	**Reference**
Water	Pure water [24,25,28,41,44,50,52,53,56,66,69,70,71,73,74,75,76]
	DEPC-water [10]
TE	[27,31,33,36,38,39,42,43,65]
Tris-HCl	[40,67]
Tris-KCl	[9,37,45]
NaOH	[68]
NaHCO_3_	[26,58]

**Table 4:** List of pathogens detected with the described LOC.

Specimens	NA	Pathogens	Reference
Blood	DNA	*Hepatitis B virus*	[23,41,54,55,60,61]
		*Bacillus subtilis, Escherichia coli*	[28,34,41,62]
		*Plasmodium falciparum*	[32,40]
		*Mycobacterium tuberculosis*	[53,63]
		*Orientia tsutsugamushi*	[58]
		*Staphylococcus warneri, Streptococcus agalactiae, Haemophilus influenzae*	[64]
	RNA	*Rift Valley fever virus*	[28]
		*Human immunodeficiency virus*	[40,61]
		*SFTS virus*	[58]
		*Influenza A virus*	[69,72]
Mucosal lining or Nasopharyngeal fluids	DNA	*Bacillus anthracis*	[25]
		*Bordetella pertussis*	[44]
		*Mycobacterium tuberculosis*	[53,63]
		*Staphylococcus aureus*	[56,68]
		*Bacillus cereus*	[66]
	RNA	*Human immunodeficiency virus*	[66]
		*Parainfluenza virus, Rhinovirus A, Metapneumovirus*	[30]
		*Respiratory-syncytial virus*	[30,70]
		Multiple respiratory RNA viruses	[71]
		*SARS-CoV-2*	Table 5
Urine	DNA	*Staphylococcus aureus*	[56]
		*Escherichia coli*	[75]
Stools	DNA	*Clostridium difficile*	[27,74]
		*Helicobacter pylori*	[73]
	RNA	*Human Enterovirus 71*	[27]
Spinal fluids	DNA	*Herpes simplex virus*, *Varicella zoster virus*	[43]

**Table 5:** List of patented and authorized POC for SARS-CoV-2 detection.

COVID-19 POC Test	Sample	Time	LoD	Reference
Xpert Xpress	300 µL	45 min	100 copies/mL	[109,110,111]
ePlex	200 µL	90 min	1000 copies/mL	[110]
Novodiag	250 µL	75 min	313 copies/mL	[111]
ID NOW	200 µL	13 min	125 copies/mL	[112]
Accula Test	10 µL	30 min	200 copies/mL	[113,114]
FilmArray	300 µL	60 min	330 copies/mL	[115]
Vivalytic	300 µL	39 min	n.a.	[116]

## Supplementary Materials

The following are available online at https://www.mdpi.com/article/10.3390/s21093058/s1, Table S1: Off-Chip Sample Preparation.
