# Supplementary material for: An Overview on Microfluidic Systems for Nucleic Acids Extraction from Human Raw Samples
Source: Sensors (Basel). 2021 Apr 27;21(9):3058. doi: 10.3390/s21093058 (PMC8125272; doi:10.3390/s21093058)
Supplement: Supplementary file 1 [file sensors-21-03058-s001.zip › sensors-1161407-supplementary.pdf]

OFF-CHIP SAMPLE PREPARATION			
**CHEMICAL LYSIS**			
**Sample type**	**Off-chip sample pretreatment**	**Lysis type**	**Reference**
Whole blood, 50 μL	Load solution preparation (50 μL): Sample (50 μL), 30% Triton X-100 (17 μL), Proteinase K (30 μL; at 20 mg/mL) and loading buffer (403 μL) containing 6M GuHCl, 10 mM Tris, 1 mM EDTA, pH 6.0. Mixing for 30 sec.	Chemical	33
Whole blood, 4 μL<br>Nasal Aspirate, 8 μL	Load solution preparation (100 μL): Sample (4 or 8 μL), Proteinase K (5 μL), 6M GuHCl (91 or 87 μL). Vortexing for 30 sec.	Chemical	44
Semen, 1.5 μL	Load solution preparation (200 μL): Sample (1.5 μL), 6M GuHCl with 40mM DDT (198.5 μL). Vortexing.	Chemical	76
Whole blood, 4 μL<br>Semen, 5 μL<br>Buccal/nasal swab, 10 μL	Load solution preparation: Crude sample or cells in water solution (4/10/5/10 μL), Proteinase K (10/20/0/10 μL), GuHCl (486/500/495/180 μL) added of 40mM DDT in case of semen. Vortexing for 15 sec.	Chemical	25
Whole blood, 10 μL<br>Spinal fluid, 40 μL	Load solution preparation (1000 μL): Sample (10 or 40 μL), 10% or 1% Triton X-100 (54 μL or 60 μL). Vortexing for 5 min. Reaching the final volume by addition of 6M GuHCl. Filtration with 0.22 μm sterile filter before loading.	Chemical	43
Whole blood, 50 μL	Load solution preparation: Sample (50 μL) pipetted for 5 min with GuSCN-based lysis buffer (RLT Buffer, Qiagen) at room temperature	Chemical	48
Whole blood, 20 μL	Load solution preparation (400 μL): Sample (20 μL); Proteinase K (20 μL),1% Triton X-100 (160 μL), NaCl and water (200 μL).	Chemical	37
Whole blood, 0.1 - 100 μL<br>Throat swabs, 300 μL	Load solution preparation: Blood (0.1 to 100 μL) mixed with GuSCN-based lysis buffer (350 - 700 μL; Buffer RLT, Qiagen). Throat swabs solution (300 μL) mixed with lysis/binding buffer (350 μL; 100mM Tris-HCl, 500mM LiCl, 10mM EDTA, 1% LiDS, 5mM DDT). Load solution is prepared by addition of oligo-dT magnetic beads, mixed and incubated at room temperature for 5 min, before charging on syringe pumps.	Chemical	30
Whole blood	Load solution preparation: Whole blood, with cancer cells, mixed with GuSCN-based lysis buffer (Buffer RLT, Qiagen) and pressure-vacuum delivered across the membrane.	Chemical	47
Nasal wash, 30 μL	Load solution preparation: Sample (30 μL) homogenized by hand passage into a 25-gauge needle for 5 times. Infected sample is mixed with lysis/binding buffer (300 μL; 2M GuSCN, 25mM sodium citrate, pH 7, 50% ethanol) and silica coated magnetic beads (20 μL). Mixed for 5 min at room temperature.	Chemical	70
Nasal wash/swab, 100 μL	Load solution preparation: Sample (100 μL) mixed with lysis buffer (300 μL; 2M GuSCN). Lysed solution is added with a syringe pump.	Chemical	69
Semen, 25 μL	Load solution preparation: Sample (4 μL) is mixed with lysis buffer (96 μL; 6M GuHCl with 40mM DDT) and loaded onto the chip with a syringe pump.	Chemical	10
Whole Blood, 5 μL	Load solution preparation: Sample (5 μL) mixed with lysis buffer (45 μL; 50mM MES, 1% Triton X-100 and 2 mg/μL of Proteinase K) and incubated for 30 min at room temperature. Lysed solution (50 μL) loaded with a manual syringe.	Chemical	45
Whole Blood, 90 μL	Load solution preparation: sample (90 μL) is mixed with lysis buffer (GuSCN-based), detergents (N-lauroylsarcosine, 2-mercaptoethanol, NP-40) and incubated for 15 min at room temperature.	Chemical	40
**MECHANICAL + CHEMICAL LYSIS**			
Whole blood, 10 μL	DNA pre-concentration on lysate: lysis buffer (125 μL; 6M GuHCl) loaded onto the octadecyl (C18) reversed-phase pre-column. Sample (10 μL) is then loaded. DNA is pre-concentrated and eluted from this phase, which then binds proteins with stronger affinity than DNA, as a sieve.	Mechanical<br>Chemical	38
Stool, 180-220 mg	Sample (180 - 220 mg) mixed and incubated with 5.5M GuSCN (700 μL) for 30 min. Lysed sample filtered on a 0.2 μm pore-size filter prior the extraction to ensure the correct fluid circulation and prevent a blockage by solid pieces. Liquid stool samples are well processed by this device without the use of electrical energy but, in case of solid stool samples, it would be necessary to centrifuge the sample before the use.	Mechanical<br>Chemical	74

Table S1: Pretreatment procedures applied to different sample types when an off-chip lysis was performed prior the loading on LOC. Similarly to the described on-chip lysis procedures (Table2), guanidinium salts (GuHCl and GuSCN), often coupled with detergents, are the most used chaotropic agents with the capacity of solubilizing proteins.

10 Hagan, K.A.; Bienvenue, J.M.; Moskaluk, C.A.; Landers, J.P. Microchip-based solid-phase purification of RNA from biological samples. *Anal. Chem.* **2008**, *80*, 8453–8460, doi:10.1021/ac8011945.

25 Legendre, L.A.; Bienvenue, J.M.; Roper, M.G.; Ferrance, J.P.; Landers, J.P. A simple, valveless microfluidic sample preparation device for extraction and amplification of DNA from nanoliter-volume samples. *Anal. Chem.* **2006**, *78*, 1444–1451, doi:10.1021/ac0516988.

30 Han, N.; Shin, J.H.; Han, K.H. An on-chip RT-PCR microfluidic device, that integrates mRNA extraction, cDNA synthesis, and gene amplification. *RSC Adv.* **2014**, *4*, 9160–9165, doi:10.1039/c3ra47980c.

33 Wu, Q.; Jin, W.; Zhou, C.; Han, S.; Yang, W.; Zhu, Q.; Jin, Q.; Mu, Y. Integrated glass microdevice for nucleic acid purification, loop-mediated isothermal amplification, and online detection. *Anal. Chem.* **2011**, *83*, 3336–3342, doi:10.1021/ac103129e.

37 Kim, J.; Gale, B.K. Quantitative and qualitative analysis of a microfluidic DNA extraction system using a nanoporous AlOx membrane. *Lab Chip* **2008**, *8*, 1516–1523, doi:10.1039/b804624g.

38 Wen, J.; Guillo, C.; Ferrance, J.P.; Landers, J.P. Microfluidic-based DNA purification in a two-stage, dual-phase microchip containing a reversed-phase and a photopolymerized monolith. *Anal. Chem.* **2007**, *79*, 6135–6142, doi:10.1021/ac0703698.

40 Kolluri, N.; Albarran, N.; Fan, A.; Olson, A.; Sagar, M.; Young, A.; Gomez-Marquez, J.; Klapperich, C.M. SNAPflex: A paper-and-plastic device for instrument-free RNA and DNA extraction from whole blood. *Lab Chip* **2020**, *20*, 3386–3398, doi:10.1039/d0lc00277a.

43 Wu, Q.; Bienvenue, J.M.; Hassan, B.J.; Kwok, Y.C.; Giordano, B.C.; Norris, P.M.; Landers, J.P.; Ferrance, J.P. Microchip-based macroporous silica sol-gel monolith for efficient isolation of DNA from clinical samples. *Anal. Chem.* **2006**, *78*, 5704–5710, doi:10.1021/ac060390t.

44 Easley, C.J.; Karlinsey, J.M.; Bienvenue, J.M.; Legendre, L.A.; Roper, M.G.; Feldman, S.H.; Hughes, M.A.; Hewlett, E.L.; Merkel, T.J.; Ferrance, J.P.; et al. A fully integrated microfluidic genetic analysis system with sample-in-answer-out capability. *Proc. Natl. Acad. Sci. U. S. A.* **2006**, *103*, 19272–19277, doi:10.1073/pnas.0604663103.

45 Cao, W.; Easley, C.J.; Ferrance, J.P.; Landers, J.P. Chitosan as a polymer for pH-induced DNA capture in a totally aqueous system. *Anal. Chem.* **2006**, *78*, 7222–7228, doi:10.1021/ac0603911.

47 Kokoris, M.; Nabavi, M.; Lancaster, C.; Clemmens, J.; Maloney, P.; Capadanno, J.; Gerdes, J.; Battrell, C.F. Rare cancer cell analyzer for whole blood applications: Automated nucleic acid purification in a microfluidic disposable card. *Methods* **2005**, *37*, 114–119, doi:10.1016/j.ymeth.2005.07.002.

48 Lee, H.; Jung, J.; Han, S.I.; Han, K.H. High-speed RNA microextraction technology using magnetic oligo-dT beads and lateral magnetophoresis. *Lab Chip* **2010**, *10*, 2764–2770, doi:10.1039/c005145d.

69 Cao, Q.; Mahalanabis, M.; Chang, J.; Carey, B.; Hsieh, C.; Stanley, A.; Odell, C.A.; Mitchell, P.; Feldman, J.; Pollock, N.R.; et al. Microfluidic chip for molecular amplification of influenza a RNA in human respiratory specimens. *PLoS One* **2012**, *7*, 1–11, doi:10.1371/journal.pone.0033176.

70 Bordelon, H.; Adams, N.M.; Klemm, A.S.; Russ, P.K.; Williams, J. V.; Talbot, H.K.; Wright, D.W.; Haselton, F.R. Development of a low-resource RNA extraction cassette based on surface tension valves. *ACS Appl. Mater. Interfaces* **2011**, *3*, 2161–2168, doi:10.1021/am2004009.

74 Huang, S.; Do, J.; Mahalanabis, M.; Fan, A.; Zhao, L.; Jepeal, L.; Singh, S.K.; Klapperich, C.M. Low Cost Extraction and Isothermal Amplification of DNA for Infectious Diarrhea Diagnosis. *PLoS One* **2013**, *8*, doi:10.1371/journal.pone.0060059.

76 Bienvenue, J.M.; Legendre, L.A.; Ferrance, J.P.; Landers, J.P. An integrated microfluidic device for DNA purification and PCR amplification of STR fragments. *Forensic Sci. Int. Genet.* **2010**, *4*, 178–186, doi:10.1016/j.fsigen.2009.02.010.
